# Class I_A_ PI3Kinase Regulatory Subunit, p85α, Mediates Mast Cell Development through Regulation of Growth and Survival Related Genes

**DOI:** 10.1371/journal.pone.0028979

**Published:** 2012-01-04

**Authors:** Subha Krishnan, Raghuveer Singh Mali, Karl R. Koehler, Sasidhar Vemula, Anindya Chatterjee, Joydeep Ghosh, Baskar Ramdas, Peilin Ma, Eri Hashino, Reuben Kapur

**Affiliations:** Department of Pediatrics, Indiana University School of Medicine, Indianapolis, Indiana, United States of America; Case Western Reserve University, United States of America

## Abstract

Stem cell factor (SCF) mediated KIT receptor activation plays a pivotal role in mast cell growth, maturation and survival. However, the signaling events downstream from KIT are poorly understood. Mast cells express multiple regulatory subunits of class 1_A_ PI3Kinase (PI3K) including p85α, p85β, p50α, and p55α. While it is known that PI3K plays an essential role in mast cells; the precise mechanism by which these regulatory subunits impact specific mast cell functions including growth, survival and cycling are not known. We show that loss of p85α impairs the growth, survival and cycling of mast cell progenitors (MCp). To delineate the molecular mechanism (s) by which p85α regulates mast cell growth, survival and cycling, we performed microarray analyses to compare the gene expression profile of MCps derived from WT and p85α-deficient mice in response to SCF stimulation. We identified 151 unique genes exhibiting altered expression in p85α-deficient cells in response to SCF stimulation compared to WT cells. Functional categorization based on DAVID bioinformatics tool and Ingenuity Pathway Analysis (IPA) software relates the altered genes due to lack of p85α to transcription, cell cycle, cell survival, cell adhesion, cell differentiation, and signal transduction. Our results suggest that p85α is involved in mast cell development through regulation of expression of growth, survival and cell cycle related genes.

## Introduction

Mast cells are critical mediators of inflammation, innate immunity and host defense that originate from multipotent stem cells in the bone marrow (BM) [1]. Mast cells have been implicated in inflammatory diseases including multiple sclerosis [2], rheumatoid arthritis [3] and coronary artery disease [3] and inflammatory diseases [4]. Emerging data also suggests a crucial role for mast cells in tumor progression and angiogenesis [5].

The homing, growth, differentiation and survival of mast cell are regulated by complex network of growth factors and transcription factors. While several cytokines influence the growth, survival and maturation of mast cells, SCF and its interaction with KIT receptor are critical for normal mast cell development and function. Mice that lack either KIT or SCF are completely devoid of mature mast cells in all tissues [6], [7], [8]. However, the intracellular signals downstream from KIT in regulating both growth and survival of mast cells are poorly understood. Recent studies have shown that PI3Kinase (PI3K), which binds to tyrosine at position 719 in murine KIT (at 721 in human KIT) through its regulatory subunit contributes substantially to KIT mediated mast cell functions [9], [10]. Class IA PI3Kinase is a lipid kinase made up of p85 regulatory subunit(s) and p110catalytic subunit(s) [11], [12]. In hematopoietic cells, four regulatory (p85α, p85β, p55α and p50α) and three catalytic (p110α, p110β and p110δ) subunits of class IA PI3K are expressed. Utilizing genetic and genomic approaches, we evaluate the role of p85α in mast cell development and functions in response to SCF stimulation. We provide evidence for the critical role of p85α in mast cell growth, survival and cycling; and possible pathways by which p85α regulates mast cell functions in response to SCF stimulation.

## Results

### Deficiency of p85α results in reduced mast cell growth in response to SCF

**Figure 1:**
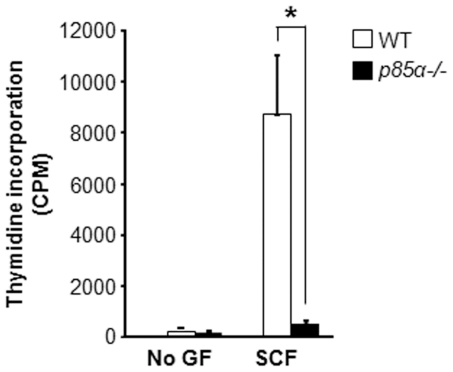
Deficiency of p85α results in reduced SCF-mediated BMMC growth. BMMCs from WT and *p85α−/−* mice were cultured in the presence of IL-3 (10 ng/mL) for 3 weeks. Cells were starved for 6 hours in serum- and cytokine-free media and cultured in the presence or absence of SCF (50 ng/mL) for 48 hours. Then, proliferation was evaluated by [^3^H] thymidine incorporation. Bars represent the mean [^3^H] thymidine incorporation in BMMCs (CPM ± SD) from one representative experiment performed in quadruplicate. Similar results were observed in three independent experiments. *p<0.01, WT vs. *p85α−/−*.

While mast cells express various regulatory subunits of Class 1A PI3K including p85α, p85β, p55α and p50α, the physiological role of these subunits in mast cell growth, survival and cycling are not known. Here we sought to evaluate the specific role of p85α in mast cell growth, survival and cycling in response to SCF stimulation. We generated *in vitro* bone marrow–derived mast cells (BMMC) from WT and *p85α−/−* mice. Loss of p85α in *p85α−/−* BMMCs was confirmed by western blotting (Data not shown). To assess the contribution of p85α in mast cell growth, BMMCs from WT and *p85α−/−* mice were subjected to proliferation assay in the presence or absence of SCF. As seen in Figure 1, BMMCs from WT and *p85α−/−* mice grown in the absence of growth factors showed minimal thymidine incorporation. While WT BMMCs demonstrated a significant increase in growth in the presence of SCF, deficiency of p85α resulted in complete loss of SCF mediated growth (Figure 1). These results suggest that the p85α regulatory subunit is critical for SCF-induced mast cell growth.

### Reconstitution of p85α into *p85α−/−* MCps completely corrects defective SCF-mediated growth

**Figure 2:**
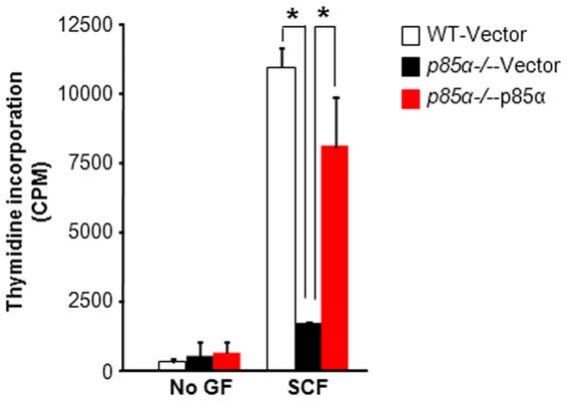
Reconstitution of p85α in to *p85α−/−* MCps completely corrects defective SCF-mediated growth. WT and *p85α−/−* MCps transduced with vector or p85α were sorted to homogeneity based on EGFP expression and cultured in the presence of IL-3 (10 ng/mL). Cells were starved for 6 hours in serum- and cytokine-free media and cultured in the presence or absence of SCF (50 ng/mL). After 48 hours, proliferation was evaluated by a [^3^H] thymidine incorporation assay. Bars represent the mean [^3^H] thymidine incorporation in BMMCs (CPM ± SD) from one representative experiment performed in quadruplicate. Similar results were observed in three independent experiments. *p<0.01, WT-vector vs. *p85α-/–*vector; *p<0.01, *p85α-/–*vector vs. *p85α-/–*p85α.

To determine whether the complete loss of SCF-mediated mast cell growth in *p85α−/−* cells is due to loss of p85α, we transduced mast cell progenitors (MCp) from WT and *p85α−/−* mice with vector or p85α and cells were sorted to homogeneity based on EGFP expression. Sorted cells were starved and analyzed proliferation in the presence or absence of SCF by thymidine incorporation. As expected, while WT cells transduced with vector showed increase in growth in the presence of SCF, vector transduced *p85α−/−* cells showed complete loss of growth (Figure 2). As seen in Figure 2, restoring the expression of p85α in p85α-deficient MCps completely restored SCF-induced growth. These results suggest that the defective SCF-mediated growth observed in *p85α−/−* BMMCs is due to specific loss of p85α.

### Defective survival of p85α-deficient BMMCs in response to SCF

**Figure 3:**
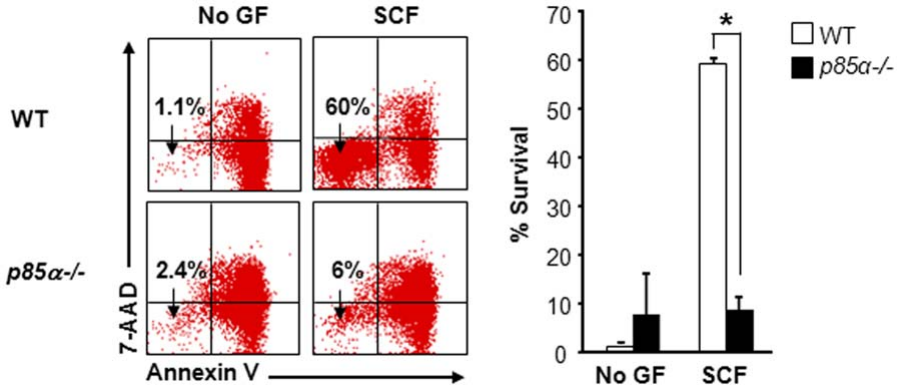
Defective survival of p85α-deficient BMMCs. BMMCs from WT and *p85α−/−* mice were starved for 6 hours in serum- and cytokine-free media and cultured in the presence or absence of SCF (50 ng/mL). After 48 hours, cells were stained with phycoerythrin-conjugated annexin V and 7-AAD followed by flow cytometry analysis. Shown is a representative dot blot (Left panel) and bar graph (Right panel) demonstrating percentage of annexin V and 7-AAD negative cells in the presence and absence of SCF. Similar results were observed in three independent experiments. *p<0.05, WT vs. *p85α−/−*.

In an effort to further determine the mechanism(s) behind reduced SCF-mediated growth of *p85α−/−* cells, BMMCs from WT and *p85α−/−* mice were subjected to apoptosis analysis in the presence or absence of SCF. BMMCs from WT and *p85α−/−* mice were starved for 6 hours in serum- and cytokine-free media and grown in the presence or absence of SCF. After 48 hours, apoptosis was assessed by staining the cells with antibodies to annexin V and 7-AAD followed by flow cytometry analysis. As seen in Figure 3, less than 10% of BMMCs from WT and *p85α−/−* mice survived in the absence of growth factors as determined by the presence of annexin V and 7-AAD negative cells. While WT BMMCs showed significant increase in survival in the presence of SCF, deficiency of p85α resulted in significant reduction in SCF-mediated survival (Figure 3). These results suggest that p85α acts as a positive regulator of mast cell survival in response to SCF.

### Reduced cycling of p85α-deficient BMMCs in response to SCF

**Figure 4:**
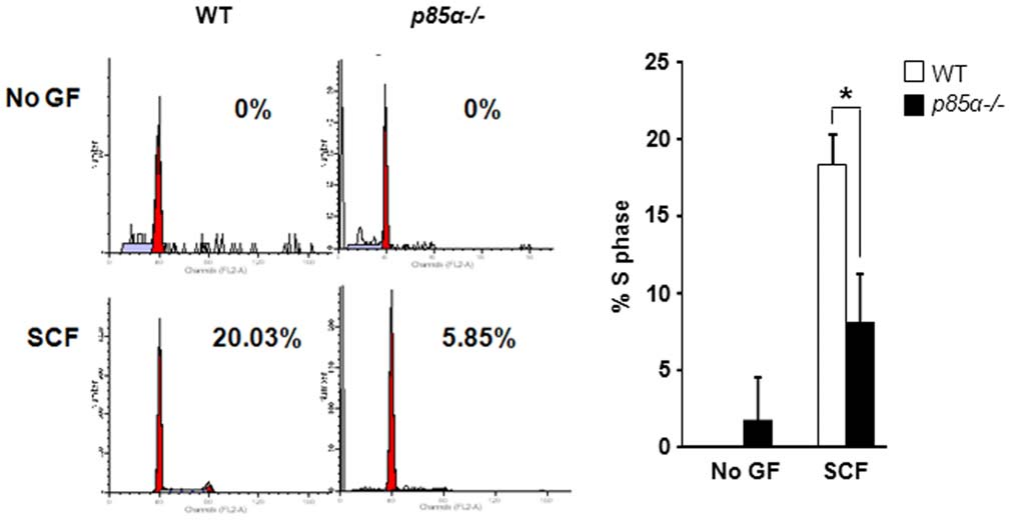
Reduced cycling of p85α-deficient BMMCs. BMMCs from WT and *p85α−/−* mice were starved for 6 hours in serum- and cytokine-free media and cultured in the presence or absence of SCF (50 ng/mL). After 48 hours, cells were stained with propidium iodide followed by flow cytometry analysis as described in methods. Shown is a representative histogram (Left panel) and bar graph (Right panel) demonstrating the percentage of cells in S phase in the presence or absence of SCF. Similar results were observed in two independent experiments. *p<0.05, WT vs. *p85α−/−*.

We then examined cell cycle progression in WT and *p85α−/−* BMMCs in response to SCF stimulation. BMMCs from WT and *p85α−/−* mice were starved for 6 hours in serum- and cytokine-free media and grown in the presence or absence of SCF. After 48 hours, cells were labeled with propidium iodide and cycling was analyzed by flow cytometry. As seen in Figure 4, while SCF stimulation enhanced the cycling of WT BMMCs, *p85α−/−* cells showed impaired cell cycle progression and S phase entry. These results suggest that p85α plays a critical role in cycling of mast cells in response to SCF.

### Deficiency of p85α alters the expression of genes related to growth and survival in MCps

**Figure 5:**
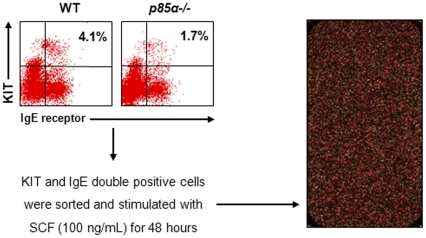
Methodology followed for microarray analysis. Low density mononuclear cells (LDMNC) were isolated from three pairs of WT and *p85α−/−* mice and cultured independently in the presence of IL-3 (10 ng/mL) for 1 week. KIT and IgE receptor double positive mast cells were sorted by using FACS. Sorted mast cells were stimulated with SCF (100 ng/mL) for 48 hours and then sent for microarray analysis for gene expression profiling.

Our results suggest that p85α subunit plays a critical role in proliferation, survival and cycling of BMMCs. To further determine the underlying molecular mechanism for the action of p85α on growth and survival, we performed microarray analyses to compare the gene expression profile of MCps derived from WT and p85α-deficient mice when stimulated with SCF (Figure 5). To determine significantly altered genes in p85α-deficient MCps compared to WT, we calculated the *p* value for gene expression pattern based on Student's t-test using Partek software with the following criteria: change in expression of at least 1.5-fold and p<0.05. This analysis identified 151 unique genes exhibiting altered expression in *p85α−/−* cells (Table S1). Raw data and normalized expression data are available for download from Gene Expression Omnibus (http://ncbi.nlm.nih.gov/geo, accession number GSE32410).

**Figure 6:**
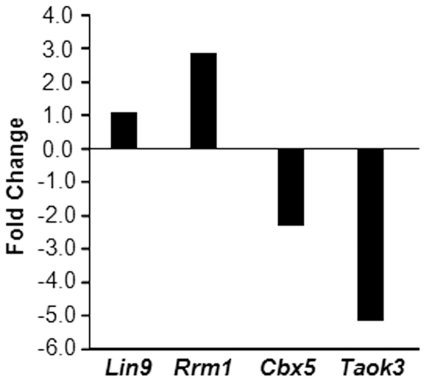
Validation of microarray analysis. Low density mononuclear cells (LDMNC) were isolated from WT and *p85α−/−* mice and cultured in the presence of IL-3 (10 ng/mL) for 1 week. KIT and IgE receptor double positive mast cells were sorted by using FACS. Sorted mast cells were stimulated with SCF (100 ng/mL) for 48 hours and mRNA was extracted using the RNeasy Plus Mini Kit (Qiagen, Valencia, CA). Qualitative real-time PCR was performed for the indicated genes using primers from Applied Biosystems as described in methods. Results show fold change of indicated genes, normalized to *Actin*, in p85α-deficient MCps compared to WT MCps.

To validate the microarray results, we analyzed the expression of four important genes (*Lin9, Rrm1, Cbx5, and Taok3*) in MCps derived from WT and p85α-deficient mice when stimulated with SCF by quantitative real-time PCR analysis. As seen in Figure 6, consistent with our microarray results, *Lin9* and *Rrm1* genes were up regulated 1.06 and 2.85 fold, respectively, in p85α-deficient MCps compared to WT MCps. Likewise, *Cbx5* and *Taok3* genes were down regulated 2.35 and 5.19 fold, respectively, in p85α-deficient MCps compared to WT MCps.

**Figure 7:**
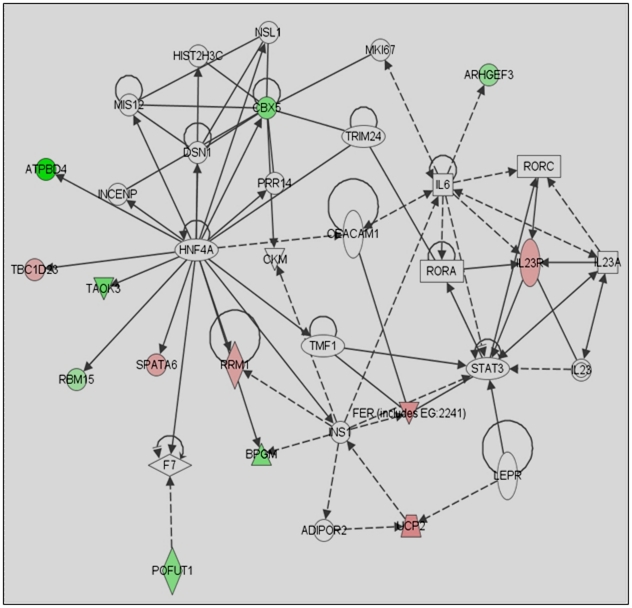
Ingenuity Pathway Analysis (IPA) of cell growth related genes in *p85α−/−* MCp's in response to SCF stimulation. Altered genes in *p85α^−/−^* cells after SCF stimulation were analyzed by IPA software to analyze their closely associated gene network. Significantly altered cell growth related genes in *p85α−/−* MCp's in response to SCF stimulation were indicated.

**Figure 8:**
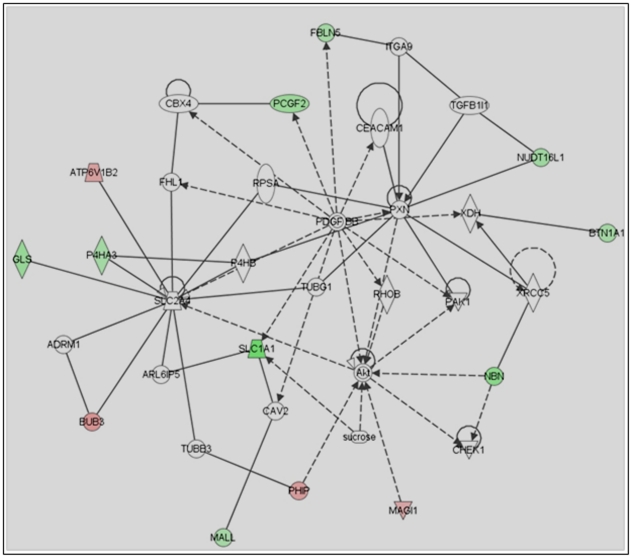
Ingenuity Pathway Analysis (IPA) of cell survival related genes in *p85α−/−* MCp's in response to SCF stimulation. Altered genes in *p85α^−/−^* cells after SCF stimulation were analyzed by IPA software to analyze their closely associated gene network. Significantly altered cell survival related genes in *p85α−/−* MCp's in response to SCF stimulation were indicated.

**Figure 9:**
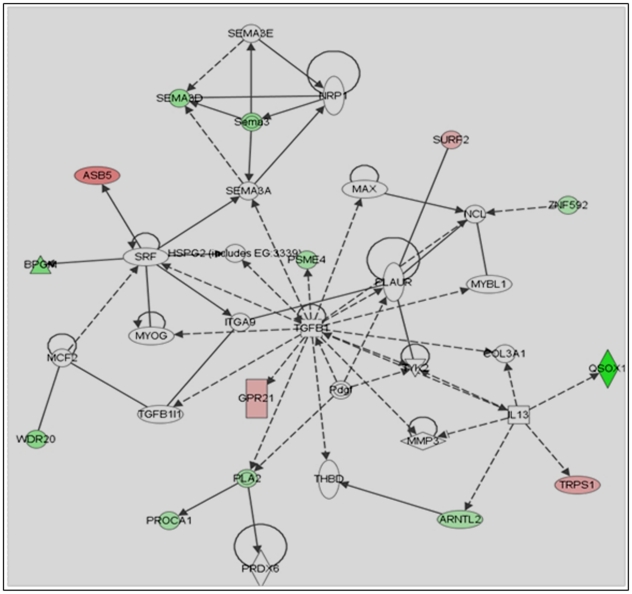
Ingenuity Pathway Analysis (IPA) of cell cycle related genes in *p85α−/−* MCp's in response to SCF stimulation. Altered genes in *p85α^−/−^* cells after SCF stimulation were analyzed by IPA software to analyze their closely associated gene network. Significantly altered cell cycle related genes in *p85α−/−* MCp's in response to SCF stimulation were indicated.

To further make the microarray data more meaningful in the context of how they alter mast cell biology, we functionally categorized these genes based on the DAVID bioinformatics tool, and Ingenuity Pathway Analysis (IPA) software. David tool relates altered genes due to lack of p85α to transcription, cell cycle, cell adhesion, cell differentiation, cell survival and signal transduction (Table 1 and Table 2). Ingenuity Pathway Analysis (IPA) software associated altered gene in *p85α−/−* with cell growth (Figure 7), cell survival (Figure 8), and the cell cycle (Figure 9) networks. Identification of these four principal networks is relevant to this study, as our functional data suggest that mast cells lacking the p85α regulatory subunit display defective growth, altered survival and differentiation (see below). The IPA network provides further information on additional genes including *Pdgfb*, *Akt*, *Hnf4A*, *Stat3*, *Tgfb*, *Myc*, *Brca1*, *Crebbp* (Figure 6, 7, 8). These genes identified by IPA tool, which are related to the altered genes in *p85α−/−* per the microarray data, could also play roles in mast cell biological functions in response to SCF stimulation. Genetic disruption of p85α resulted in upregulation of tumor suppressor gene *Lin9* (Lin-9 homolog); and *Rrm1* (Ribonucleotide Reductase M1), which is reported to induce the expression of PI3K negative regulator PTEN [13]. Of the down regulated genes - *Nsd1*, which is related to acute myeloid leukemia and prostate cancer [14]; Tgfb-inducible *Fbln5*, which are important in cell growth, migration and cell adhesion [15]; *Taok3* (Tao kinase 3), which has been reported to activate ERK1/ERK2 [16]; and *Pim3* (Pim3 kinase) which has been reported to positively regulate cell proliferation and survival, down regulation of which might contribute significantly to increased apoptosis in p85α-deficient cells are of significance for future studies [17].

**Table 1:** Functional categories of genes upregulated in *p85α−/−* MCp's in response to SCF stimulation.

Functional Category	Gene Symbol	Gene Name	Fold Change
**Ubl conjugation pathway**	Senp6	Sumo/Sentrin Specific Peptidase 6	1.62
	Asb5	Ankyrin Repeat And Socs Box-Containing protein	2.80
	Ubr2	Riken Cdna E130209G04 gene	1.84
**Proteolysis**	Senp6	Sumo/Sentrin Specific Peptidase 6	1.62
	Ubr2	Riken Cdna E130209G04 gene	1.84
	Klk6	Kalikrein 6	1.92
	Rnf150	Ring Finger Protein 150	4.39
**Cell Surface receptor**	Il23r	Interleukin 23 Receptor	1.74
	Olfr1107	Olfactory Receptor 1107	1.73
	Sufu	Suppressor of Fused Homolog	2.11
	Olfr1179	Olfactory Receptor 1179	1.58
	Gpr21	G Protein-Coupled Receptor 21	1.57
**Cell cycle**	Bub3	Budding uninhibited by Benzimidazoles 3 homolog	20.9
	Ubr2	Riken Cdna E130209G04 gene	1.84
	Lin9	Lin-9-Homolog (C. Elegans)	4.16
	Cetn1	Centrin 1	2.63
	Atp6v1b2	Atpase, H+ Transporting, Lysosomal V1 Subunit B2	1.67
	Rrm1	Ribonucleotide Reductase M1	1.67
	Agps	Alkylglycerone Phosphate Synthase	2.30
	Cetn1	Centrin 1	2.63

**Table 2:** Functional categories of genes downregulated in *p85α−/−* MCp's in response to SCF stimulation.

Functional Category	Gene Symbol	Gene Name	Fold Change
**Transcription Regulation**	Nsd1	Nuclear receptor-binding set domain protein 1	−2.11
	Arntl2	Aryl hydrocarbon receptor nuclear translocator-like	−1.72
	Pcgf2	Polycomb group ring finger 2	−1.90
	Tcf20	Transcription factor 20	−3.50
	Pou4f3	Pou domain, class 4, transcription factor 3	−1.55
	Uimc1	Retinoid x receptor interacting protein 110	−3.06
**Protein Kinase Activity**	Pim3	Proviral integration site 3	−3.63
	Taok3	Tao kinase 3	−3.41
	Dyrk1a	Dual-specificity tyrosine phosphorylation regulated kinase 1a	−2.13
**Cell differentiation**	Sema3d	Riken cDNA 4631426b19 gene	−2.26
	Psme4	Proteasome activator subunit 4	−1.77
	Mbnl1	Muscle blind-like 1 (drosophila)	−2.03
	Pou4f3	Pou domain, class 4, transcription factor 3	−1.55
**Cell adhesion**	Cdh8	Cadherin 8	−7.09
	Fbln5	Fibulin 5	−1.64
	Col2a1	Procollagen, type ii alpha 1	−1.56
**Signal transduction**	Pofut1	Protein o-fucosyl transferase 1	−2.66
	Arntl2	Aryl hydrocarbon receptor nuclear translocator-like 2	−1.72
	Taok3	Tao kinase 3	−3.41
	Arhgef3	Rho guanine nucleotide exchange factor 3	−2.18
	Rabl2a	Rab, member of ras oncogene family-like 2a	−1.58
**Post-translational protein modification**	Nsd1	Nuclear receptor binding set domain protein 1	−2.11
	Pim3	Proviral integration site 3	−3.63
	Taok3	Tao kinase 3	−3.41
	Pcgf2	Polycomb group ring finger 2	−1.90
	Fbxo3	F-box only protein 3	−1.90
	Dyrk1a	Dual-specificity tyrosine phosphorylation regulated kinase 1a	−2.13

## Discussion

Emerging data have demonstrated the crucial role of SCF-mediated KIT signaling in mast cell development [8], [18], [19], [20], [21]. Downstream from KIT receptor, PI3Kinase (PI3K) plays a critical role in mast cell development and its functions including growth, survival, differentiation, adhesion and migration [10]. PI3K is a complex of regulatory (p85) and catalytic subunits (p110), which gets activated upon binding to KIT receptor via its regulatory subunit. Current dogma in PI3K is that the primary function of regulatory subunits of class I_A_ PI3K is mainly to stabilize and activate the different p110 catalytic subunits. Recent studies have also shown the involvement of PI3K in abnormal KIT signaling due to activating KIT mutation (KITD816V in human and KITD814V in mouse), which causes hematologic malignancies including mastocytosis and mast cell leukemia [22], [23].

In this study, we evaluated the role of p85α regulatory subunit in mast cell growth, survival and cycling. We showed that genetic disruption of p85α results in defective SCF-mediated mast cell growth, survival and cycling. The defective SCF-mediated mast cell growth was observed irrespective of the presence of other PI3K regulatory subunits including p85β, p55α and p50α suggesting that p85α might play a unique role in SCF-mediated mast cell growth. These results were further supported by the fact that reconstitution of *p85α−/−* BMMCs with p85α completely corrects KIT-induced proliferation. These findings suggest that the defective SCF-mediated growth observed in *p85α−/−* BMMCs is a result of specific loss of p85α and not due to quantitative reduction in the overall expression of regulatory subunits.

To further identify the molecular mechanism (s) by which p85α regulates mast cell growth, survival and cycling, we performed microarray analyses to compare the gene expression profile of MCps derived from WT and p85α-deficient mice in response to SCF stimulation. We identified 151 unique genes that were altered in p85α-deficient MCps compared to WT MCps in response to SCF stimulation. Ingenuity pathway analysis was performed on these 151 altered genes, which revealed some interesting pathways that are effected due to deficiency of p85α including AKT and PDGF pathway which is important in cell growth and survival [24], [25]; IL-6, important in cell growth [26]; and Myc which is important in cell growth and survival [27]; TGF-β pathway which is important in cell migration [28]. Furthermore, the deficiency of p85α also enhances apoptotic signals in mast cells by upregulating genes involved in proteolysis and UBL conjugation pathway, which might be partly responsible for significantly reduced proliferation in response to SCF stimulation. Moreover, altered expression of cell cycle genes Rrm1 and Taok3 could also be important in regulating mast cell growth. Rrm1 is a negative regulator of PI3K activity through induction of PTEN expression [29], the expression of which is enhanced in absence of p85α regulatory subunit. Taok3 is an enhancer of ERK activity [16], which is down regulated in absence of p85α. Thus p85α might directly or indirectly via. HNF4A regulate the expression of these genes in response to SCF stimulation. Moreover, p85α might regulate mast cell migration via. TGF-β pathways as indicated by IPA result.

These findings have high significance in clinical implications as mast cells are increasingly recognized as key components in regulating tumor progression as well as inflammatory diseases including multiple sclerosis [2], rheumatoid arthritis [3] and coronary artery disease [3] and inflammatory diseases [4]. Previous studies have also shown increased mast cell infiltration with worse prognosis in many human cancers [30], [31]. This study provides insight into proteins that regulate SCF-mediated mast cell growth, survival, and cycling, which can be further explored to develop future therapeutic treatments to human diseases involving mast cells.

## Materials and Methods

### Cytokines, Antibodies and Reagents

Recombinant murine interleukin-3 (IL-3) and stem cell factor (SCF) were purchased from Pepro Tech (Rocky Hill, NJ). Phycoerythrin (PE)-conjugated KIT antibody, fluorescence isothyocyanate (FITC)–conjugated IgE receptor antibody, PE-conjugated annexin V antibody and 7-Amino actinomycin D (7-AAD) were purchased from BD Biosciences (San Jose, CA). Rabbit anti-p85 pan antibody (clone UB93-3) and mouse anti-p85α–specific antibody (clone AB6) were purchased from Upstate Biotechnology Inc., (Lake Placid, NY). Retronectin was purchased from Takara (Madison, WI). Iscove's Modified Dulbecco's Medium (IMDM) was purchased from Invitrogen (Carlsbad, CA). [^3^H] thymidine was purchased from PerkinElmer (Boston, MA).

### Mice

*p85α−/−* mice have been previously described_ENREF_28 [32]. All mice were maintained under specific pathogen-free conditions at the Indiana University Laboratory Animal Research Center (Indianapolis, IN). All studies were approved by the Indiana University Laboratory Animal Resource Center (Study # 3137 and Study # 937).

### RNA isolation and cDNA preparation from murine spleen

Cells were harvested from wild-type C57BL/6 mice by flushing out the spleen using IMDM media and the low density cells were collected after density gradient centrifugation using Histopaque 1083. Total RNA was isolated from these cells using QIAGEN RNeasy Kit. Total RNA isolated was quantified and also purity was measured using spectrophotometer. cDNA for RT-PCR was prepared from 5 µg of total RNA using SuperScript First-Strand Synthesis System as per instructions (Invitrogen, Carlsbad, CA). 1 µg of cDNA was used to isolate gene of interest by PCR amplification with specific primers (see below).

### Construction of p85α subunit

RNA was isolated and cDNA was prepared from murine spleen. Following synthesis of cDNA, the following primers were used for p85α PCR: forward, 5′-GAATTCATGTACCCATACGATGT TCCAGATTACGCTATGAGTGCAGAGGGCTACCAG; reverse, 5′-CTCGAGTCATCGCCTCTG TTGTGCATATAC. Restriction sites used for cloning purposes have been underlined. PCR was performed using the following conditions: an initial denaturation step at 94°C for 2 min followed by 23 cycles of 94°C for 30 s, 60°C for 1 min, and 72°C for 2 min, with a final step of 72°C for 7 min. The plasmid have a HA tag at the amino terminus and was cloned into the EcoRI/XhoI site upstream of an internal entry site and the enhanced green fluorescence (EGFP) protein containing bi-cistronic retroviral vector MIEG3.

### Preparation of retroviral supernatants for transduction

Retroviral supernatants for transduction of primary mast cell progenitors (MCp) were generated using the Phoenix ecotropic packaging cell line (provided by Dr. Garry Nolan, Stanford University, Stanford, CA) transfected with retroviral vector plasmids using a calcium phosphate transfection kit (Invitrogen, Carlsbad, CA) [33]. Supernatants were collected after 48 hours of transfection and filtered through 0.45 uM membranes.

### Generation of *in vitro* bone marrow derived mast cells (BMMC) from wildtype (WT) and *p85α−/−* mice

To generate BMMCs, low density mononuclear cells (LDMNC) were isolated from WT and *p85α−/−* mice and cultured in IMDM supplemented with 10% FBS, 2% penicillin/streptomycin and 10 ng/mL of IL-3 for 3–4 weeks. These cells were used at different stages for growth, survival and cell cycle experiments.

### Expression of vector or p85α into mast cell progenitors (MCp)

To express vector or p85α subunit in MCps, LDMNCs were collected from WT and *p85α−/−* mice, and pre-stimulated in IMDM supplemented with 20% FBS, 2% penicillin/streptomycin, and cytokines (100 ng/mL SCF and 10 ng/mL IL-3) for 48 hours prior to retroviral infection on fibronectin fragments (Retronectin) in non-tissue culture plates. On third day, MCps were infected with 4 mL of high-titer retroviral supernatants for vector or p85α prepared as described above. A second shot of viral infection was given twenty four hours later. Forty-eight hours after the second infection, cells expressing EGFP were sorted and utilized to perform all experiments.

### Proliferation assay

Cell proliferation was assessed by conducting a thymidine incorporation assay as described [34]. Briefly, cells were washed twice with warm IMDM and starved in IMDM supplemented with 0.2% BSA for 6 to 7 hours. 5×10^4^ cells were plated in a 96-well plate in 200 µl of IMDM supplemented with 10% fetal bovine serum plus 2% penicillin/streptomycin in the presence or absence of SCF. Cells were cultured for 48 hours and subsequently pulsed with 1.0 µCi of [^3^H] thymidine for 6 to 8 hours at 37°C. Cells were harvested using an automated 9-well cell harvester (Brandel; Gaithersburg, MD) and thymidine incorporation was determined as counts per minute (CPM).

### Apoptosis and Cell Cycle

To determine the cell cycle and apoptosis, cells were washed 2 times with IMDM to remove any serum or cytokines. The cells were then starved for 7 hours in IMDM supplemented with 0.2% BSA. Then, 0.2×10^6^ cells were cultured in the presence or absence of SCF (100 ng/mL). After 48 hours, cells were washed with PBS and the percentage of cell death was determined by annexin V and 7-AAD staining. Cells were re-suspended in 1× annexin V binding buffer and incubated for 30 min at 4°C. Cells were incubated with 5 µL each of annexin V and 7-AAD for 20 min at room temperature in the dark. After incubation, percentage of apoptotic cells (annexin V and 7-AAD positive) was determined using flow cytometry. For cell cycle analysis, cells were labeled by propidium iodide (PI) and analyzed by flow cytometry.

### Sample preparation for microarray analysis

LDMNCs were harvested from 3 independent WT and *p85α−/−* mice and cultured in IMDM supplemented with 10% fetal bovine serum, 2% penicillin/streptomycin, and 10 ng/mL of IL-3 for 7 days. Cells were collected by centrifugation, resuspended in 200 µL PBS containing 0.2% BSA, and then incubated with 5 µg of anti-PE-KIT and anti-FITC-IgE antibody for 30 min at 4°C. Cells were washed with PBS containing 0.2% BSA to remove any unbound antibodies. The mast cell population that demonstrated double expression of KIT and IgE receptors was then sorted and collected. The sorted cells were then stimulated with SCF (100 ng/mL) for 48 hours, after which, they were pelleted, frozen immediately and stored at −80°C until further use for microarray analysis.

### Microarray processing and data analysis

Frozen mast cells stimulated with SCF (100 ng/mL) from 3 independent WT and *p85α−/−* mice (as described above) were sent to Miltenyi Biotech to carry out the microarray analysis. Due to smaller sample size (<10,000 cells), super-amplification technology was used for the analysis. This amplification was based on a global PCR protocol using mRNA-derived cDNA. mRNA was isolated *via* magnetic bead technology. Amplified cDNA from the mRNA samples was quantified and quality was evaluated by capillary electrophoresis on an Agilent 2100 Bioanalyzer platform (Agilent Technologies; Santa Clara, CA). 250 ng of WT and *p85α−/−* cDNAs was labeled with fluorescent dye cy3 or cy5 and simultaneously hybridized overnight (17 hours, 65°C) to an Agilent whole-mouse genome oligo microarray 4×44 K. In order to avoid differential incorporation of the 2 dyes into target samples, dye incorporation rate was carefully monitored. To avoid breakdown of cy5 by ozone, an ozone-free microarray facility in Miltenyi Biotech was maintained with specially installed ozone extractors. Subsequently, microarrays were washed and signals were detected using Agilents DNA microarray scanner. Probe-set intensities were obtained and a quantile normalization procedure was used to adjust for the difference in probe intensity distribution across different chips. For our analysis, we included genes that showed a fold-change >1.5 and *p*<0.05.

### Quantitative PCR analysis

Total RNA was isolated using the RNeasy Minikit (Qiagen) and then treated with TURBO DNase (Ambion) to remove genomic DNA. Single-stranded cDNA was synthesized using Omniscript reverse transcriptase (Qiagen) according the manufacturer's instructions. Gene expression was analyzed on an ABI PRISM 7900HT Sequence Detection System (Applied Biosystems) using TaqMan Gene Expression Assays (Applied Biosystems) and the FastStart Universal Probe Master Mix (Roche). For data analysis, ΔCT values were calculated using ß-Actin (*Actb*) expression as an internal control. Fold change between wild-type and p85α samples was calculated using the 2^(ΔΔCT)^ method. Data are representative of three technical replicates for one biological sample. Expression was principally confirmed by two biological replicates.

### Statistics

All graphical data was evaluated by paired Student *t-* test and results were considered significantly different with *p*-value <0.05. All data are represented as mean values ± standard deviations (SD).

## Supporting Information

Table S1 **List of genes altered in** ***p85α−/−*** **MCp's in response to SCF stimulation.** Low density mononuclear cells (LDMNC) were isolated from three pairs of WT and *p85α−/−* mice and cultured independently in the presence of IL-3 (10 ng/mL) for 1 week. KIT and IgE receptor double positive mast cells were sorted by using FACS. Sorted mast cells were stimulated with SCF (100 ng/mL) for 48 hours and then sent for microarray analysis for gene expression profiling. Altered expression of genes in *p85α−/−* MCp's in response to SCF stimulation compared to WT controls were listed in Table S1.
(DOC)
