# Supplementary material for: Class IA PI3Kinase Regulatory Subunit, p85α, Mediates Mast Cell Development through Regulation of Growth and Survival Related Genes
Source: PLoS One. 2012 Jan 4;7(1):e28979. doi: 10.1371/journal.pone.0028979 (PMC3251560; doi:10.1371/journal.pone.0028979)
Supplement: Table S1 — List of genes altered in p85α−/− MCp's in response to SCF stimulation. Low density mononuclear cells (LDMNC) were isolated from three pairs of WT and p85α−/− mice and cultured independently in the presence of IL-3 (10 ng/mL) for 1 week. KIT and IgE receptor double positive mast cells were sorted by using FACS. Sorted mast cells were stimulated with SCF (100 ng/mL) for 48 hours and then sent for microarray analysis for gene expression profiling. Altered expression of genes in p85α−/− MCp's in response to SCF stimulation compared to WT controls were listed in Table S1. (DOC) [file pone.0028979.s001.doc]

**Table S1. List of genes altered in *p85α-/-* MCp’s in response to SCF stimulation.**

| **Gene Symbol** | **Gene description** | **Fold Change** | **p-Value** |
| --- | --- | --- | --- |
| 4930447A16Rik | RIKEN cDNA 49300447A16 gene | -7.69 | 0.02 |
| Cdh8 | Cadherin 8 | -7.09 | 0.05 |
| Atpbd4 | ATP binding domain 4 | -6.37 | 0.008 |
| 1810047C23Rik | RIKEN cDNA 18104723C23 gene | -612 | 0.03 |
| Qsox1 | Quiescin Q6 sulfhydryl oxidase 1 | -5.58 | 0.03 |
| A030006P16Rik | Keratin associated protein 17-1 | -5.21 | 0.024 |
| NAP069078-1 | NAP069078-1 | -4.64 | 0.001 |
| 1700017D01Rik | 1700017D01Rik | -4.63 | 0.01 |
| AK047500 | AK047500 | -4.5 | 0.007 |
| Laptm4t | Lysosomal-associated protein transmembrane 4B | -4.13 | 0.005 |
| 9330155M09Rik | RIKEN cDNA 9330155M09 gene | -3.85 | 0.02 |
| Txndc5 | Thiroedoxin domain containing 4 | -3.72 | 0.001 |
| Pim3 | Proviral integration site 3 | -3.64 | 0.000904 |
| Tcf20 | Transcription factor 20 | -3.5 | 0.01 |
| Taok3 | TAO kinase 3 | -3.41 | 0.02 |
| Tc1531727 | TC1531727 | -3.38 | 0.02 |
| 4922502D21Rik | RIKEN cDNA 4922502D21 gene | -3.33 | 0.02 |
| Ubl4b | Ubiquitin-like 4B | -3.32 | 0.03 |
| Sic1a1 | Solute carrier family 1 | -3.28 | 0.05 |
| D330028D13Rik | RIKEN cDNA D330028D13 gene | -3.15 | 0.01 |
| 1700069L16Rik | RIKEN cDNA 1700069L16 gene | -3.09 | 0.02 |
| Uimc1 | Ubiquitin interaction motif containing 1 | -3.06 | 0.02 |
| Cbx5 | Chromobox homolog 5 | -2.95 | 0.04 |
| Bpgm | 2.3-bisphosphoglycerate mutase | -2.95 | 0.03 |
| 1200009106Rik | RIKEN cDNA 1200009106 gene | -2.93 | 0.02 |
| Dus 1l | Dihydrouridine synthase 1-like | -2.86 | 0.02 |
| A_52_P868798 | A_52_p868798 | -2.98 | 0.03 |
| Pofut1 | Protein O-fucosyltransferase 1 | -2.67 | 0.04 |
| D830039M14Rik | RIKEN cDNA D830039M14 gene | -2.58 | 0.04 |
| BC049715 | Hypothetical protein LOC100044248 | -2.54 | 0.02 |
| AB182283 | AB182283 | -2.48 | 0.05 |
| 2310040A13Rik | WD repeat domain 20A | -2.44 | 0.02 |
| Nbn | Nibrin | -2.41 | 0.02 |
| Olfr414 | Olfactory receptor 414 | -2.31 | 0.03 |
| NAP123291-1 | NAP123291-1 | -2.28 | 0.04 |
| ENSMUST00000101328 | ENSMUST00000101328 | -2.28 | 0.03 |
| Sema3d | Sema domain, immunoglobulin domain | -2.26 | 0.04 |

**Table S1. List of genes altered in *p85α-/-* MCp’s in response to SCF stimulation (con’t).**

| **Gene Symbol** | **Gene description** | **Fold Change** | **p-Value** |
| --- | --- | --- | --- |
| 1700023L04Rik | RIKEN cDNA 1700023L04 gene | -2.21 | 0.04 |
| Sic25a10 | Solute carrier family 25, member 10 | -2.21 | 0.03 |
| Arthgef3 | Rho guanine nucleotide exchange factor (GEF)3 | -2.19 | 0.05 |
| 1700021K19Rik | RIKEN cDNA 1700021K19 gene | -2.18 | 0.03 |
| Dyrk1a | Dual-specificity tyrosine-(Y)-phosphorylationregulated kinase 1a | -2.13 | 0.04 |
| Nsd1 | Nuclear receptor-binding SET-domain protein 1 | -2.12 | 0.03 |
| Abca12 | ATP-binding cassette, sub-family A (ABC1) | -2.11 | 0.02 |
| Defb41 | Defensin beta 41 | -2.11 | 0.02 |
| Deaf1 | Deformed epidermal autoregulatory factor 1 | -2.10 | 0.02 |
| D930016D06Rik | RIKEN cDNA D930016D06 gene | -2.10 | 0.02 |
| Mbnl1 | Muscleblind-like 1 | -2.04 | 0.009 |
| AK087625 | AK087625 | -2.04 | 0.04 |
| Stard3 | START domain containing 3 | -2.03 | 0.02 |
| Zfp667 | Zinc finger protein 667 | -2.02 | 0.04 |
| Gls | Glutaminase | -1.97 | 0.04 |
| Cain1 | Calneuron 1 | -1.92 | 0.04 |
| Fbxo3 | F-box protein 3 | -1.91 | 0.04 |
| Pcgf2 | Polycomb group ring finger 2 | -1.90 | 0.02 |
| F730031O20Rik | Cas scaffolding protein family member 4 | -1.88 | 0.02 |
| ENSMUST00000061772 | ENSMUST00000061772 | -1.86 | 0.009 |
| AK085452 | Predicted gene 9973 | -1.84 | 0.017 |
| Nudt16l1 | Nudix type motif 16-like 1 | -1.77 | 0.02 |
| Psme4 | Proteasome activator subunit 4 | -1.77 | 0.04 |
| 4933404M19Rik | RIKEN cDNA 4933404M19 gene | -1.77 | 0.04 |
| Rp2h | Retinitis pigmentosa 2 homolog | -1.75 | 0.02 |
| Rsad2 | Radical S-adenosyl methionine domain containing 2 | -1.74 | 0.02 |
| Amtl2 | Aryl hydrocarbon receptor nuclear translocator | -1.73 | 0.01 |
| LOC620807 | LOC620807 | -1.72 | 0.04 |
| Mov10l1 | Moloney leukemia virus 10-like 1 | -1.70 | 0.04 |
| Pex11c | Peroxisomal biogenesis factor 11 gamma | -1.70 | 0.03 |
| Zdhhc5 | Zinc finger, DHHC domain containing 5 | -1.68 | 0.02 |
| Olfr309 | Olfactory receptor 309 | -1.66 | 0.01 |
| Zfp592 | Zinc finger protein 592 | -1.66 | 0.02 |
| Btn1a1 | Buytophilin, subfamily 1 member A1 | -1.65 | 0.005 |
| Fbln5 | Fibulin 5 | -1.64 | 0.02 |
| 2310021P13Rik | RIKEN cDNA 2310021P13 gene | -1.64 | 0.02 |
| Ubie | Methyltransferase like 7A2 | -1.63 | 0.04 |
| P4ha3 | Procollagen-proline, 2-oxoglutarate 4-dioxygenase alpha polypeptide III | -1.56 | 0.03 |

**Table S1. List of genes altered in *p85α-/-* MCp’s in response to SCF stimulation (con’t).**

| **Gene Symbol** | **Gene description** | **Fold Change** | **p-Value** |
| --- | --- | --- | --- |
| Mobkl1a | MOB1, Mps One Binder kinase activator-like 1A | 1.77 | 0.01 |
| Smgc | Submandibular bland protein C | 1.79 | 0.04 |
| AA536717 | AA536717 | 1.79 | 0.04 |
| AK033099 | AK033099 | 1.79 | 0.01 |
| Phip | Pleckstrin homology domain interacting protein | 1.82 | 0.01 |
| Ubr2 | Ubiquitin protein ligase E3 component n-recognin 2 | 1.85 | 0.05 |
| Trps1 | Trichorhinophalangeal syndrome l | 1.87 | 0.02 |
| Phox2a | Paired-like homeobox 2a | 1.88 | 0.03 |
| Sptlc1 | Serine palmitoyltransferase, long chain base subunit 1 | 1.92 | 0.01 |
| AK033617 | AK033617 | 1.92 | 0.007 |
| Klk6 | Kallikrein related-peptidase 6 | 1.93 | 0.02 |
| Prdm4 | PR domain containing 4 | 1.95 | 0.01 |
| Lyrm5 | LYR motif containing 5 | 1.95 | 0.04 |
| AK019507 | AK019527 | 1.97 | 0.01 |
| Bub3 | Budding uninhibited by benzimidazoles 3 homolog | 2.10 | 0.01 |
| Sufu | Suppressor of fused homolog | 2.10 | 0.01 |
| Fert2 | Fer (fms/fps related) protein kinase, testis specific 2 | 2.25 | 0.006 |
| 2610020C11Rik | Zinc finger protein 157 | 2.26 | 0.03 |
| AK082639 | AK082639 | 2.30 | 0.01 |
| Agps | Alkylglycerone phosphate synthase | 2.30 | 0.04 |
| Ift81 | Intraflagellar transport 81 homolog | 2.42 | 0.03 |
| Ucp2 | Uncoupling protein 2 | 2.43 | 0.02 |
| AK047379 | AK047379 | 2.45 | 0.003 |
| A630007B06Rik | RIKEN cDNA A630007B06 gene | 2.45 | 0.03 |
| D930043O14Rik | RIKEN cDNA D930043O14 gene | 2.51 | 0.04 |
| Cetn1 | Centrin 1 | 2.64 | 0.05 |
| Hisppd2a | Histidine acid phosphatase domain containing 2A | 2.66 | 0.02 |
| Asb5 | Ankyrin repeat and SOCs box-containing 5 | 2.80 | 0.01 |
| Kcnj14 | Potassium inwardly-rectifying channel, subfamily | 2.88 | 0.03 |
| 4930413O22Rik | RIKEN cDNA 4930413O22 gene | 3.34 | 0.006 |
| AK082897 | AK083897 | 3.48 | 0.01 |
| Acad11 | Acyl-Coenzyme A dehydrogenase family | 3.90 | 0.02 |
| Lin9 | Lin-9 homolog | 4.17 | 0.03 |
| Zfp26 | Zinc finger protein 26 | 4.30 | 0.006 |
| Rnf150 | Ring finger protein 150 | 4.40 | 0.01 |
| NAP028759-1 | NAP028759-1 | 4.43 | 0.005 |
| 2610016C23Rik | Family with Sequence similarity 54, member A | 4.58 | 0.01 |
| Ttc29 | Tetratricopeptide repeat domain 29 | 4.65 | 0.002 |
| 1110032A13Rik | RIKEN cDNA 1110032A13 gene | 6.83 | 0.04 |
